# Implementation of Antibiotic Stewardship in a University Hospital Setting

**DOI:** 10.3390/antibiotics10010093

**Published:** 2021-01-19

**Authors:** Milan Kolar, Miroslava Htoutou Sedlakova, Karel Urbanek, Patrik Mlynarcik, Magdalena Roderova, Kristyna Hricova, Kristyna Mezerova, Pavla Kucova, Jana Zapletalova, Katerina Fiserova, Pavel Kurfurst

**Affiliations:** 1 Department of Microbiology, University Hospital Olomouc, Faculty of Medicine and Dentistry, Palacký University Olomouc, 779 00 Olomouc, Czech Republic; milan.kolar@fnol.cz (M.K.); patrik.mlynarcik@upol.cz (P.M.); magdalena.roderova@upol.cz (M.R.); kristyna.hricova@upol.cz (K.H.); kristyna.mezerova@upol.cz (K.M.); pavla.kucova@fnol.cz (P.K.); katerina.fiserova@fnol.cz (K.F.); 2 Department of Pharmacology, University Hospital Olomouc, Faculty of Medicine and Dentistry, Palacký University Olomouc, 779 00 Olomouc, Czech Republic; karel.urbanek@fnol.cz; 3 Department of Medical Biophysics, Faculty of Medicine and Dentistry, Palacký University Olomouc, 779 00 Olomouc, Czech Republic; ja.zapletalova@upol.cz; 4 Department of Foreign Languages, Faculty of Medicine and Dentistry, Palacký University Olomouc, 779 00 Olomouc, Czech Republic; pavel.kurfurst@upol.cz

**Keywords:** antibiotic stewardship, resistance, consumption of antibiotics, clonal spread

## Abstract

The article describes activities of an antibiotic center at a university hospital in the Czech Republic and presents the results of antibiotic stewardship program implementation over a period of 10 years. It provides data on the development of resistance of *Escherichia coli*, *Klebsiella pneumoniae*, *Pseudomonas aeruginosa* and *Staphylococcus aureus* to selected antibiotic agents as well as consumption data for various antibiotic classes. The genetic basis of resistance to beta-lactam antibiotics and its clonal spread were also assessed. The study showed significant correlations between aminoglycoside consumption and resistance of *Escherichia coli* and *Klebsiella pneumoniae* to gentamicin (r = 0.712, r = 0.869), fluoroquinolone consumption and resistance of *Klebsiella pneumoniae* to ciprofloxacin (r = 0.896), aminoglycoside consumption and resistance of *Pseudomonas aeruginosa* to amikacin (r = 0.716), as well as carbapenem consumption and resistance of *Pseudomonas aeruginosa* to meropenem (r = 0.855). Genotyping of ESBL- positive isolates of *Klebsiella pneumoniae* and *Escherichia coli* showed a predominance of CTX-M-type; in AmpC-positive strains, DHA, EBC and CIT enzymes prevailed. Of 19 meropenem-resistant strains of *Klebsiella pneumoniae*, two were identified as NDM-positive. Clonal spread of these strains was not detected. The results suggest that comprehensive antibiotic stewardship implementation in a healthcare facility may help to maintain the effectiveness of antibiotics against bacterial pathogens. Particularly beneficial is the work of clinical microbiologists who, among other things, approve administration of antibiotics to patients with bacterial infections and directly participate in their antibiotic therapy.

## 1. Introduction

Antibiotic stewardship may be defined as a set of measures leading to rational antibiotic therapy based on the adequate selection of antibacterial agents, appropriate duration of their administration and a suitable route of administration [1,2,3,4]. The need for antibiotic stewardship implementation stems from the likely prospect of antibiotics losing their effectiveness and thus their ability to treat bacterial infections [5,6,7]. The increasing prevalence of bacteria resistant to antibacterial drugs, mainly those producing extended-spectrum beta-lactamases including metallo-beta-lactamases and carbapenemases opens the possibility of a new non-antibiotic era in which adequate antibiotics will be unavailable to treat infections caused by multidrug-resistant bacteria [8,9]. To prevent this, antibiotic stewardship programs have been developed as comprehensive systems comprising a range of activities that may be briefly characterized as follows:

- early and adequate microbiological diagnosis including the correct interpretation of microbiological results,
- early and reliable detection of the susceptibility/resistance of bacterial pathogens to antibiotics consistent with the European guidelines, namely those by the European Committee on Antimicrobial Susceptibility Testing (EUCAST) [10],
- immediate reporting of critical results (e.g., information on positive blood cultures),
- regular assessment of the prevalence of pathogenic bacteria and their antibiotic resistance and development of local guidelines for initial antibiotic therapy based on these data,
- adequate antibiotic prophylaxis.

It must be stressed, however, that the scope of antibiotic stewardship is much broader, involving numerous other activities that are also very important for adequate antibiotic therapy and preventing the spread of multidrug-resistant bacteria. These activities may be described as follows:

- analyzing the routes of spread of multidrug-resistant bacteria using modern molecular methods,
- providing antibiotic consultant service for clinical physicians and deciding on antibiotic administration based on microbiological results and the knowledge of primary resistance of bacterial pathogens in patients with bacterial infections,
- assessing the consumption of antibiotics in the relevant epidemiological units and, if needed, introducing necessary regulatory measures,
- close cooperation with hospital hygiene officers, epidemiologists and clinical pharmacologists.

At the University Hospital Olomouc, Czech Republic, antibiotic stewardship is coordinated by the Antibiotic Center, a section of the Department of Microbiology. Based on analyses of the development of bacterial resistance and antibiotic consumption, including the overall costs of this group of drugs, recommendations for initial antibiotic therapy and prophylaxis are formulated and quarterly presented to the hospital management who subsequently approve these recommendations and make them valid.

The article describes efforts of the Antibiotic Center and presents outcomes of its activity over a period of 10 years (2010–2019). 

## 2. Materials and Methods

### 2.1. Characteristics of the Healthcare Facility

The University Hospital Olomouc is one of the largest inpatient healthcare facilities in the Czech Republic, dating back to 1896. It is part of a network of nine teaching hospitals directly controlled by the Ministry of Health of the Czech Republic. Basic data on the facility are shown in Table 1.

### 2.2. Process of Approving Antibiotic Administration

To better understand the study, it is reasonable to define the process of approving antibiotic administration at the University Hospital Olomouc. For a particular patient with a bacterial infection, the attending physician selects an antibiotic based on their own clinical reasoning and microbiological results (if available), while observing the hospital’s guidelines for initial antibiotic therapy. Alternatively, an adequate antibiotic is recommended by a clinical microbiologist based on a consultation with the attending physician. If an antibiotic is selected to treat a particular bacterial infection, its administration must be approved by an Antibiotic Center member. The approval is granted electronically using the hospital information system. The clinical microbiologist (always holding a specialist qualification in medical microbiology) verifies the selection of the antibiotic focusing on all microbiological test results and, if adequate, approves its administration. The Antibiotic Center member has the right to disapprove administration of an antibiotic in case:

- some required data are missing (e.g., diagnosis of infection or antibiotic dosage),
- they reasonably doubt that the antibiotic has been adequately selected,
- ongoing microbiological tests have identified bacteria whose definitive susceptibility is yet to be determined but due to their primary resistance to the selected antibiotic, this cannot be approved.

In case of disapproval, the reason and a more adequate antibiotic or recommendations from a consultation with the Antibiotic Center clinical microbiologist are entered into the hospital information system. This takes place daily between 7 a.m. and 4 p.m. Outside these hours, antibiotic therapy is selected in line with the hospital’s guidelines and the antibiotic therapy is scrutinized on the following day.

### 2.3. Assessing Antibiotic Consumption

A computerized database of the hospital’s Department of Pharmacology was used to obtain data on antibiotic consumption during the study period. The data were processed according to the 2020 ATC/DDD system and expressed as numbers of defined daily doses for various antibiotic classes [11]. Antibiotic consumption was analyzed for both the entire hospital and its Department of Anesthesiology and Intensive Care Medicine with 25 intensive care beds.

### 2.4. Identification of Bacteria and Determination of Their Susceptibility/Resistance to Antibacterial Agents

Bacterial pathogens (*Escherichia coli*, *Klebsiella pneumoniae*, *Pseudomonas aeruginosa*, and *Staphylococcus aureus*) were isolated from clinical samples (tracheal secretion, bronchoalveolar lavage fluid, sputum, blood, urine, pus, puncture samples, wound secretion, bile, cerebrospinal fluid) obtained from hospitalized patients with a suspected bacterial infection. For each patient, only the first isolate from particular clinical samples was included.

The identification of bacteria was performed by MALDI-TOF MS (Biotyper Microflex, Bruker Daltonics, Bremen, Germany) [12].

The susceptibility/resistance to antibiotics was tested using a broth microdilution method according to the EUCAST [10]. The following reference strains were used as quality control organisms: *Escherichia coli* ATCC 25922, *Pseudomonas aeruginosa* ATCC 27853 and *Staphylococcus aureus* ATCC 29213. All strains of *Staphylococcus aureus* were also tested for the resistance to methicillin using selective diagnostic chromogenic media (Colorex/TM/MRSA, TRIOS, Prague, Czech Republic) and an immunochromatographic assay for the detection of PBP2a (PBP2a SA Culture Colony Test, Alere^TM^, Abbott, Prague, Czech Republic). The production of beta-lactamases, such as ESBL and AmpC, was detected by phenotypic tests [13]. The production of carbapenemases was detected by the Carba NP test [14].

Additionally, methicillin-resistant *Staphylococcus aureus* (MRSA) strains isolated from the Department of Anesthesiology and Intensive Care Medicine patients were confirmed by the *mecA* gene detection [15]. The production of ESBL and AmpC beta-lactamases in *Escherichia coli* and *Klebsiella pneumoniae* was confirmed by PCR detection of the *bla* genes only in pre-defined groups of strains/patients from above mentioned department (from tracheal aspirates in patients with hospital-acquired pneumonia, from stool in hospitalized patients etc.) [13]. The search for potential production of carbapenemases in the meropenem-resistant *Klebsiella pneumoniae* strains at this department was carried out by simplex PCR with primers targeting *bla*_FRI_, *bla*_GES_, *bla*_GIM_, *bla*_IMI_, *bla*_IMP_, *bla*_KPC_, *bla*_NDM_, *bla*_VIM_, *bla*_OXA-23_ and *bla*_OXA-48_. Detailed information on the primers is listed in Table 2. PCR assays were performed on Rotor-Gene TM 6000 (Corbett Research, Mortlake, Australia). PCR was run in a final volume of 25 µL using 100 ng of DNA template, 0.5 μM of forward and reverse primers, 200 μM of each dNTP, 2.5 mM of MgCl_2_ and 1.25 U Combi Taq Polymerase (Top-Bio, Vestec, Czech Republic) in 1× Buffer (Top-Bio, Vestec, Czech Republic). The PCR conditions were as follows: initial denaturation at 94 °C for 3 min, followed by 30 cycles at 94 °C for 30 s, 72 °C for different times (45 s to 60 s) with a final extension at 72 °C for 10 min. PCR products were then separated on a 1% agarose gel containing SYBR Safe (Invitrogen) and visualized on a UV transilluminator. Bacterial isolates for genetic analysis were stored in cryotubes at −80 °C (Cryobank B, ITEST, Hradec Králové, Czech Republic).

### 2.5. Clonality

The clonality of MRSA and meropenem-resistant isolates of *Klebsiella pneumoniae* detected at the Department of Anesthesiology and Intensive Care Medicine was assessed with pulsed-field gel electrophoresis (PFGE). Bacterial DNA extracted with a technique described by Husičková et al. [19] was digested by the *Xba*I restriction endonuclease (New England Biolabs, Ipswitch, MA, USA) for 24 h at 37 °C in *Klebsiella pneumoniae* isolates and by the *Sma*I restriction endonuclease (New England Biolabs, Ipswitch, MA, USA) for 24 h at 25 °C in *Staphylococcus aureus* strains. The obtained DNA fragments were separated by PFGE on 1.2% agarose gel for 24 h at 6 V/cm and pulse times of 2–35 s for both *Klebsiella pneumoniae* and *Staphylococcus aureus* strains. Subsequently, the gel was stained with ethidium bromide. The resulting restriction profiles were analyzed with the GelCompar II software (Applied Maths, Kortrijk, Belgium) using the Dice coefficient (1.2%) for comparing similarity and unweighted pair group method with arithmetic means for cluster analysis. The results were interpreted according to criteria described by Tenover et al. [20].

### 2.6. Statistical Analysis

Trends in the consumption of antibacterial agents, or antibiotic classes, bacterial resistance and their relationships were analyzed with Spearman’s correlation. The data were processed with IBM SPSS Statistics 22 (Armonk, NY, USA). 

## 3. Results

Table 3, Table 4, Table 5 and Table 6 show the prevalence of *Escherichia coli*, *Klebsiella pneumoniae*, *Pseudomonas aeruginosa* and *Staphylococcus aureus* strains resistant to selected antibiotics over the 10-year period for the entire hospital. The results indicate an increase in resistance of *Escherichia coli* to piperacillin/tazobactam (r = 0.939), gentamicin (r = 0.826), ciprofloxacin (r = 0.816) and cefotaxime (r = 0.734). In *Klebsiella pneumoniae*, resistance to ciprofloxacin (r = 0.665) and cefotaxime increased (r = 0.644). *Pseudomonas aeruginosa* was shown to increase its resistance to colistin (r = 0.722) and amikacin (r = 0.691). 

**Figure 1:**
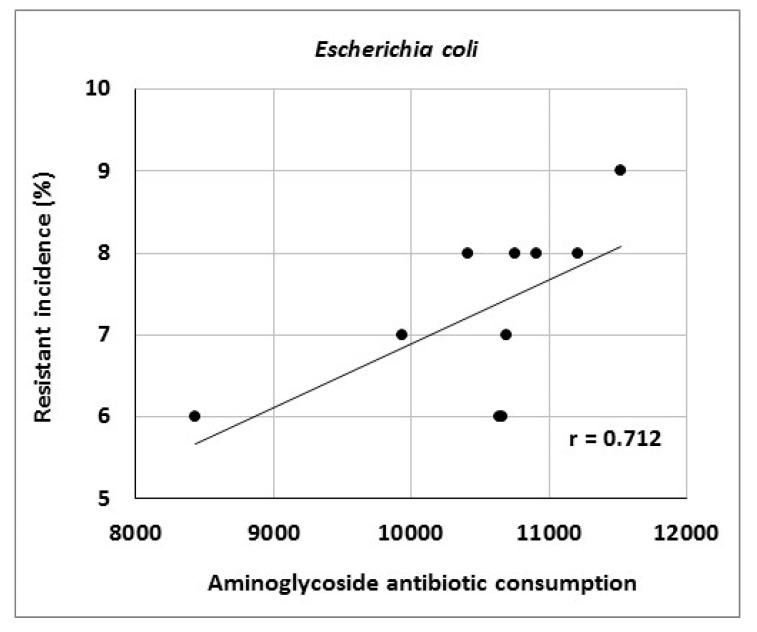
Correlation between aminoglycoside consumption (in numbers of defined daily doses) and resistance of *Escherichia coli* to gentamicin.

**Figure 2:**
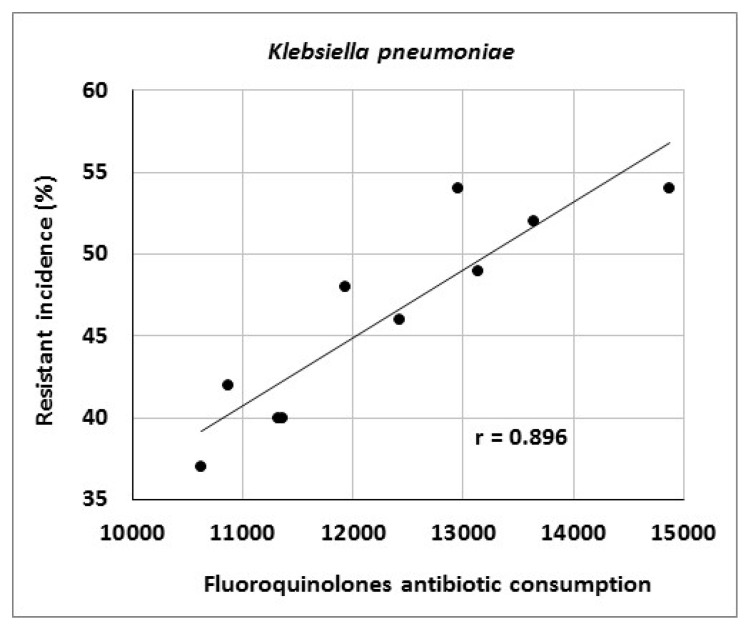
Correlation between fluoroquinolone consumption (in numbers of defined daily doses) and resistance of *Klebsiella pneumoniae* to ciprofloxacin.

**Figure 3:**
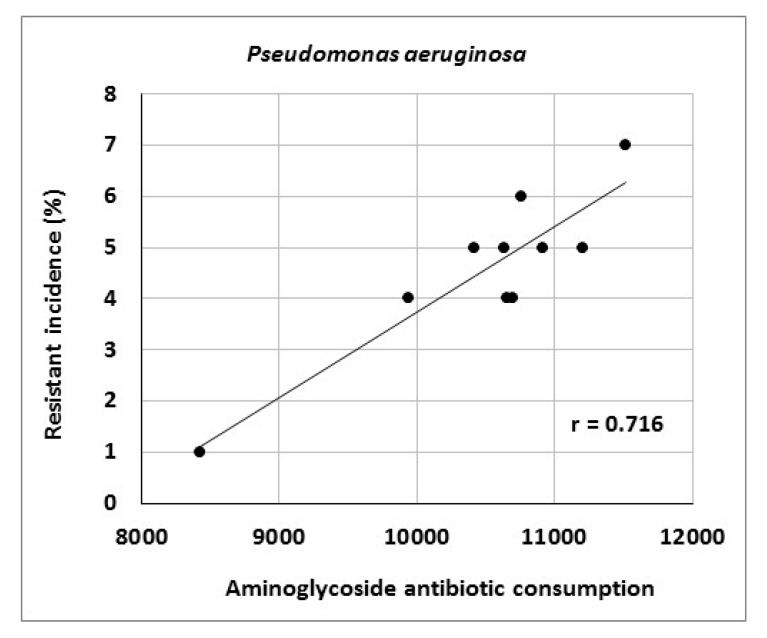
Correlation between aminoglycoside consumption (in numbers of defined daily doses) and resistance of *Pseudomonas aeruginosa* to amikacin.

Consumption of antibiotics or antibiotic classes at the University Hospital Olomouc is shown in Table 7. The data indicate increasing consumption of carbapenems (r = 0.964), tigecycline (r = 0.879), third- and fourth-generation cephalosporins (r = 0.867) and fluoroquinolones (r = 0.733). Conversely, consumption of penicillins combined with beta-lactamase inhibitors decreased (r = −0.745). Analysis of the relationship between antibiotic consumption and resistance in the entire hospital showed significant correlations between aminoglycoside consumption and resistance of *Escherichia coli* to gentamicin (r = 0.712), fluoroquinolone consumption and resistance of *Klebsiella pneumoniae* to ciprofloxacin (r = 0.896) and aminoglycoside consumption and resistance of *Pseudomonas aeruginosa* to amikacin (r = 0.716) (Figure 1, Figure 2 and Figure 3).

Table 8, Table 9, Table 10 and Table 11 document resistance of particular bacterial species at the Department of Anesthesiology and Intensive Care Medicine over the study period. The results show increasing resistance of *Escherichia coli* to piperacillin/tazobactam (r = 0.845) and cefotaxime (r = 0.729), resistance of *Klebsiella pneumoniae* to cefotaxime (r = 0.778) and resistance of *Pseudomonas aeruginosa* to meropenem (r = 0.988). 

**Figure 4:**
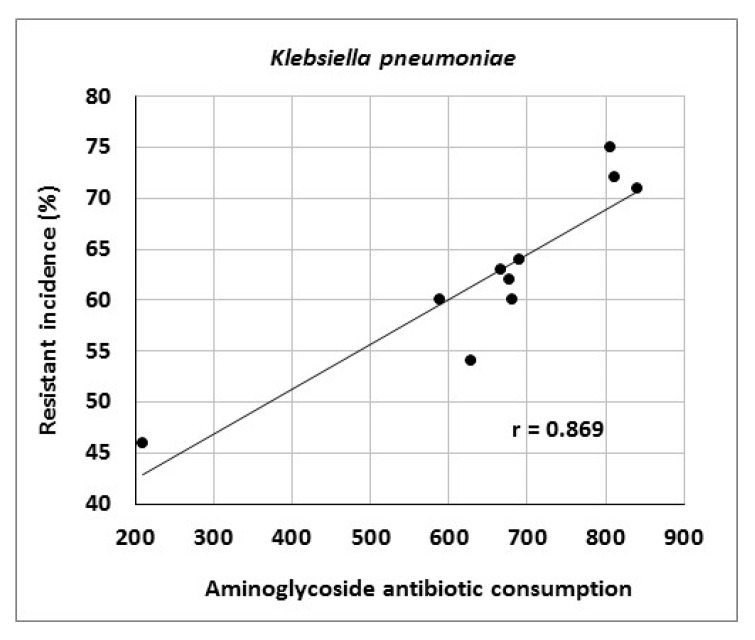
Correlation between aminoglycoside consumption (in numbers of defined daily doses) and resistance of *Klebsiella pneumoniae* to gentamicin.

**Figure 5:**
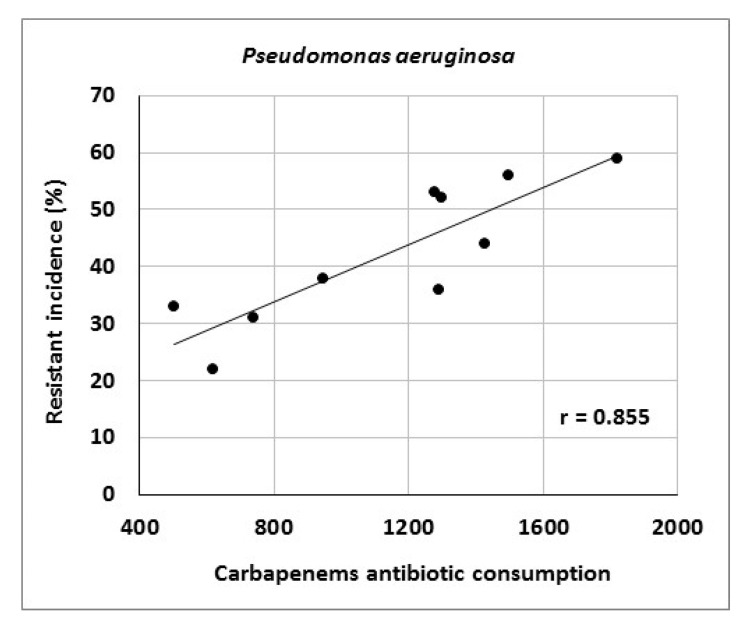
Correlation between carbapenem consumption (in numbers of defined daily doses) and resistance of *Pseudomonas aeruginosa* to meropenem.

At the Department of Anesthesiology and Intensive Care Medicine, consumption of tigecycline (r = 0.939), carbapenems (r = 0.879), third- and fourth-generation cephalosporins (r = 0.867) and glycopeptides (r = 0.636) increased (Table 12). There were significant correlations between carbapenem consumption and resistance of *Pseudomonas aeruginosa* to meropenem (r = 0.855) as well as between aminoglycoside consumption and resistance of *Klebsiella pneumoniae* to gentamicin (r = 0.869) (Figure 4 and Figure 5).

Genotyping of ESBL- positive isolates of *Klebsiella pneumoniae* and *Escherichia coli* in particular patient groups (from tracheal aspirates in patients with hospital-acquired pneumonia, from stool in hospitalized patients etc.) at the Department of Anesthesiology and Intensive Care Medicine showed a predominance of CTX-M-type, namely of the CTX-M-15 and CTX-M-9 types (data not shown). In AmpC-positive strains, EBC and CIT enzymes prevailed in *Escherichia coli* and the DHA type in *Klebsiella pneumoniae* (data not shown).

Between 2010 and 2019, a total of 19 meropenem-resistant strains of *Klebsiella pneumoniae* were detected in patients staying at the Department of Anesthesiology and Intensive Care Medicine. Only 2 strains were NDM-positive (data not shown). However, no other carbapenemase genes were detected. The total number of isolated MRSA at the Department of Anesthesiology and Intensive Care Medicine was 45 strains. In case of meropenem-resistant *Klebsiella pneumoniae* strains and MRSA, no significant clonal spread was noted. No identical clone was detected in meropenem-resistant *Klebsiella pneumoniae* isolates and only two pairs of identical MRSA strains were identified.

## 4. Discussion

Today’s medicine is characterized by exponentially expanding knowledge in all specialties, resulting in considerable improvements of both diagnostic and therapeutic activities. Despite past achievements, however, there is one issue posing a serious therapeutic challenge. It is the role of bacterial infections that have continued to increase in recent years. One reason is rising resistance of bacteria to the effects of antibacterial drugs and the associated risk of treatment failure. Numerous studies have been published documenting higher mortality and shorter survival of patients with infections caused by multidrug-resistant bacteria compared to those due to susceptible strains of the same species [21,22,23,24,25]. The present study yielded interesting results when compared with the national and European resistance rates as reported by the European Antimicrobial Resistance Surveillance Network (EARS-Net). In 2019, the mean prevalence of MRSA in the Czech Republic and Europe was 13% and 15%, respectively; the University Hospital Olomouc rates ranged from 3% to 6% [26,27]. Similarly, very low prevalence was also noted for meropenem-resistant strains of *Klebsiella pneumoniae*. According to the ECDC’s Annual Epidemiological Report for 2019, the mean prevalence of carbapenem-resistant strains of *Klebsiella pneumoniae* in Europe was 8%, with some European countries even reporting rates higher than 10% [26]. At the University Hospital Olomouc, however, the resistance of this species to meropenem did not exceed 1% or, in case of the Department of Anesthesiology and Intensive Care Medicine, 3%. Only two strains were found to produce NDM- carbapenemases. For meropenem-resistant isolates without the carbapenemase gene, we assume that the resistance is due to mechanisms such as loss or mutation of porins with AmpC beta-lactamase or ESBL hyperproduction or overexpression of the efflux pumps.

There were considerable differences in resistance of *Klebsiella pneumoniae* to third-generation cephalosporins in Europe (31%) and in the Czech Republic (50%) in 2019 [26,27]. The University Hospital Olomouc rate (43%) was below the mean rate for the entire country. 

Resistance of *Escherichia coli* to cefotaxime and resistance of *Pseudomonas aeruginosa* to ceftazidime, aminoglycosides and fluoroquinolones at the University Hospital Olomouc do not greatly differ from the mean rates in Europe. 

Of concern is the prevalence of *Pseudomonas aeruginosa* strains resistant to meropenem (34%), exceeding both the Czech (15%) and European (17%) mean rates [26,27]. However, carbapenems are mainly needed to treat infections caused by members of *Enterobacterales* producing ESBL and AmpC beta-lactamases; the resistance of these bacterial species to meropenem does not increase. Despite that, there will be efforts to reduce carbapenem consumption in the following years. It should be stated that carbapenems account for 6% of the overall antibiotic consumption at the University Hospital Olomouc (unpublished data). 

With the exception of a higher prevalence of meropenem-resistant *Pseudomonas aeruginosa*, prevalence rates of other studied phenotypes are below the rates reported by the EUCAST [26,27]. The main causes of the development and spread of bacterial resistance are the administration of antibiotics and their selection pressure [28,29,30,31,32,33,34]. Therefore, the restriction of certain antibacterial agents and relevant antibiotic classes aimed to limit their selection pressure is a possible solution to the problem [35]. However, selection pressure is a more complex issue. Apparently, consumption of certain antibiotics may only be reduced if the consumption of others increases. Moreover, antibiotic resistance is often multiple, meaning that selection pressure of a particular antibiotic agent results in increased resistance to other antibiotics, for example, resistance of ESBL-positive enterobacteria to cephalosporins and fluoroquinolones or resistance of MRSA to clindamycin [36,37]. Another important aspect influencing the selective pressure is antibiotic concentration, that is the correct dosage of antibiotics and their distribution in the body. Clinical microbiologists and physicians care about the accurate dosage in terms of pharmacodynamic/pharmacokinetic parameters to achieve satisfactory outcomes in patients. However, the question is how the selected dosage and the final concentration of an antibiotic promotes the genesis of resistant mutants. The phenomenon of bacterial resistance represents a complex problem and the emergence of antibiotic-resistant mutants depends on different aspects such physiology, genetics, historical behavior of bacterial populations, antibiotic-bacterium dynamics and others [38,39]. 

Studies have shown that there may not be a direct relationship between the administration of selected antibiotics and bacterial resistance. Several studies failed to confirm correlations between bacterial resistance to particular antibiotic classes and their consumption [40,41,42]. Similarly, Htoutou Sedláková et al. reported decreasing consumption of third-generation cephalosporins and fluoroquinolones but increasing resistance of *Enterobacteriaceae* to these drugs [43]. This may be due to multiple mechanisms. Some authors claim that the relationship between antibiotic consumption and resistance disappears after a certain resistance threshold is exceeded, since mobile genetic elements (in particular plasmids and transposons) circulate in bacterial populations and a decrease in antibiotic selection pressure does not influence this phenomenon any more [44]. It is documented that transfer rates of ESBL-plasmids are highest in the absence of the antibiotic [45]. Another explanation could be the collateral effect of antibiotics, which means that not only subinhibitory concentrations of an antibiotic could stimulate the emergence and the dissemination of its corresponding resistant gene, but that collateral stimulation by other antibiotics is also possible. For example, the mobile genetic element carrying the gene for tetracycline resistance is able to exhibit a 1000-fold increase of its transfer frequency when exposed to subinhibitory concentrations of tetracyclines, but also macrolides, lincosamides and streptogramins [46].

Our findings suggest that the increasing bacterial resistance is mainly determined by the selection pressure of antibiotics. Neither significant horizontal clonal spread of multidrug-resistant bacteria nor increasing bacterial resistance to a particular antibiotic whose consumption decreases have been observed. 

As part of antibiotic resistance surveillance, the Antibiotic Center not only controls the appropriate administration of antibiotics, that is the adequate indication and dosage in a particular patient, but also regularly monitors important bacterial resistance phenotypes and genotypes, in particular MRSA, vancomycin-resistant enterococci, ESBL- and AmpC-positive *Enterobacterales*, Gram-negative bacteria resistant to carbapenems, fluoroquinolones and others, as well as their clonal spread. For technical reasons, such surveillance is not performed in the entire hospital, but is mostly limited to selected departments and pre-defined patient groups and time periods. This approach to antibiotic stewardship has been reflected in numerous studies carried out at our department [47,48,49,50]. Based on their outcomes, certain conclusions have been drawn and relevant measures have been implemented such as evidence-based recommendations for consultant microbiologists and attending physicians concerning an adequate selection of antibiotic agents, guidelines for initial antibiotic therapy including antibiotic prophylaxis, restriction of certain antibiotic classes or improvement of hygiene and epidemiological measures.

The present study showed a significant relationship between aminoglycoside consumption and resistance of *Escherichia coli* and *Klebsiella pneumoniae* to gentamicin, results consistent with those in our 2014 study [43]. Moreover, there were correlations between fluoroquinolone consumption and resistance of *Klebsiella pneumoniae* to ciprofloxacin and between aminoglycoside consumption and resistance of *Pseudomonas aeruginosa* to amikacin, consistent with findings published by other authors [34,51]. Another reason for increasing bacterial resistance may be the horizontal or clonal spread of genetically identical strains of particular species among patients. In this case, the selection pressure of antibiotics may be of less importance and external environmental factors may play a role, for example, those related to healthcare staff. Examples include a study by Hricová et al. on vancomycin-resistant enterococci in patients with hematological malignancies at the University Hospital Olomouc reporting 67% clonality of isolated strains or outbreaks of epidemic MRSA clones in various parts of the world [48,52,53,54]. The present study, however, did not show a significant clonal spread of MRSA and meropenem-resistant strains of *Klebsiella pneumoniae* isolated from Department of Anesthesiology and Intensive Care Medicine patients, highlighting the role of horizontal resistance gene transfer in the spread of antibiotic resistance. Further, there is no doubt that the use of antibiotics contributes to the development of resistance by acquiring resistance genes and maintenance of chromosomal resistance-associated mutations [38]. However, determining the exact effect of antibiotic use on the development of resistance is problematic. Moreover, it is increasingly claimed that the emergence, maintenance and spread of resistance traits are also influenced by social, economic and genetic factors.

## 5. Conclusions

The presented data suggest low rates of bacterial resistance at the University Hospital Olomouc, with the only exception being an increased prevalence of meropenem-resistant strains of *Pseudomonas aeruginosa*. This confirms the importance of antibiotic stewardship and surveillance of antimicrobial resistance, including the use of molecular biology methods, for maintaining the effectiveness of antibiotics and limiting the spread of multidrug-resistant bacterial pathogens. Data on the prevalence of bacterial resistance and the results of molecular genetic analysis of multidrug-resistant strains must form the basis for practical antibiotic stewardship. These should include a definition of optimal regimens for initial antibiotic therapy and assessment of the sources and routes of spread of multidrug-resistant bacteria so that adequate hygiene and epidemiological measures may be introduced. It is apparent that besides obtaining data for the entire hospital, hospital departments need to be individually assessed and adequate antibiotic stewardship measures must be implemented based on the results.

## Figures and Tables

**Table 1:** Basic information on the University Hospital Olomouc in 2019.

No. of units	68
No. of beds	1198
No. of employees	4199
No. of outpatients per year	925,162
No. of inpatients per year	53,633
Mean length of stay (days)	5.6
No. of operations per year	22,715
No. of units	68

**Table 2:** Primer sequences used to detect the carbapenemase genes by PCR.

Target (Subtypes)	Primer Name	Sequence (5′ to 3′ Direction) ^a^	Amplicon Size (bp)	Tm (°C)	Reference
FRI (8)	FRI-F/R	ACAGACARGATGAGAGATTTCCT, CAGGTRCCTGTTTTATCGCC	538	58	This study
GES (42)	GES-F/R	ACGTTCAAGTTTCCGCTAG, GGCAACTAATTCGTCACGT	624	53	This study
GIM (2)	GIM-F/R	TCGACACACCTTGGTCTGAA, AACTTCCAACTTTGCCATGC	477	55	Ellington et al., 2007 [16]
IMI (11)	IMI-F/R	CTACGCTTTAGACACTGGC, AGGTTTCCTTTTCACGCTCA	482	54	Mlynarcik et al., 2016 [17]
IMP (69)	IMP-F1/R1	GAGTGGCTTAATTCTCRATC, CCAAACYACTASGTTATCT	183	52	Mlynarcik et al., 2016 [17]
IMP (2)	IMP-F2/R2	GCGGAATAGGGTGGCTTA, AGTTGCTTGGTTTTGATGGTT	435	52	This study
IMP (3)	IMP-F3/R3	TGACGGGGTTAGTTATTGGCT, CGGTTTCGCTATGACCTGAA	248	57	Mlynarcik et al., 2019 [18]
KPC (51)	KPC-F/R	GTTCTGCTGTCTTGTCTCTCA, CGGTCGTGTTTCCCTTTAG	625	56	This study
NDM (29)	NDM-F/R	GGGGATTGCGACTTATGC, AGATTGCCGAGCGACTTG	258	53	Mlynarcik et al., 2019 [18]
OXA-23-like (37)	OXA(23-like)-F/R	ACTTGCTATGTGGTTGCTTCTC, ACCTTTTCTCGCCCTTCCAT	310	56	Mlynarcik et al., 2016 [17]
OXA-48-like (31)	OXA(48-like)-F/R	AACGGGCGAACCAAGCATTTT, TGAGCACTTCTTTTGTGATGGCT	597	57	Mlynarcik et al., 2016 [17]
VIM (68)	VIM-F/R	CGCGGAGATTGARAAGCAAA, CGCAGCACCRGGATAGAARA	247	57	Mlynarcik et al., 2016 [17]

^a^ For degenerate primers: R = A or G; S = G or C; Y = C or T.

**Table 3:** Resistance of *Escherichia coli* to antibiotics at the University Hospital Olomouc in 2010–2019.

	2010	2011	2012	2013	2014	2015	2016	2017	2018	2019
**AMS**	28 (4118)	23 (3552)	28 (3558)	19 (3464)	22 (3529)	22 (3905)	25 (3998)	26 (4145)	24 (4133)	26 (4350)
**PPT**	10 (3121)	9 (2652)	11 (2624)	11 (2482)	13 (2525)	14 (2751)	16 (2953)	14 (3256)	15 (3078)	18 (3091)
**CTX**	9 (3143)	8 (2650)	13 (2624)	12 (2480)	14 (2522)	15 (2748)	16 (2950)	13 (3236)	14 (3084)	15 (3077)
**MER**	0 (3120)	0 (2651)	0 (2623)	0 (2482)	0 (2525)	0 (2750)	0 (2954)	0 (3257)	0 (3078)	0 (3091)
**GEN**	6 (4161)	6 (3552)	7 (3557)	6 (3464)	7 (3529)	8 (3908)	9 (3997)	8 (4144)	8 (4168)	8 (4378)
**AMI**	4 (3120)	3 (2651)	3 (2624)	3 (2480)	4 (2521)	3 (2751)	2 (2953)	2 (3255)	1 (3076)	1 (3091)
**CIP**	21 (3144)	18 (2651)	22 (2624)	20 (2481)	21 (2523)	21 (2754)	24 (2954)	23 (3257)	26 (3082)	27 (3093)
**COL**	0 (4129)	1 (3250)	0 (3558)	0 (3463)	1 (3529)	1 (3905)	0 (3999)	0 (4143)	0 (4147)	0 (4354)
**TIG**	1 (3080)	0 (2645)	1 (2623)	4 (2480)	2 (2520)	4 (2744)	1 (2948)	0 (3234)	1 (3079)	1 (3071)

Legend: Resistance percentages (total number of isolates tested), AMS—ampicillin/sulbactam, PPT—piperacillin/tazobactam, CTX—cefotaxime, MER—meropenem, GEN—gentamicin, AMI—amikacin, CIP—ciprofloxacin, COL—colistin, TIG—tigecycline.

**Table 4:** Resistance of *Klebsiella pneumoniae* to antibiotics at the University Hospital Olomouc in 2010–2019.

	2010	2011	2012	2013	2014	2015	2016	2017	2018	2019
**AMS**	55 (2534)	50 (1868)	46 (2017)	43 (2180)	52 (2341)	54 (2247)	52 (2189)	54 (2124)	49 (2104)	49 (2240)
**PPT**	42 (2270)	41 (1725)	39 (1821)	42 (1958)	51 (2147)	52 (2072)	52 (1986)	53 (1977)	48 (1927)	46 (2046)
**CTX**	38 (2275)	39 (1725)	37 (1821)	40 (1958)	50 (2152)	51 (2072)	51 (1988)	51 (1958)	46 (1930)	43 (2044)
**MER**	0 (2270)	<1 (1724)	<1 (1818)	<1 (1958)	<1 (2147)	0 (2069)	<1 (1986)	<1 (1975)	<1 (1926)	<1 (2047)
**GEN**	35 (2554)	36 (1867)	31 (2017)	35 (2180)	42 (2337)	45 (2247)	44 (2191)	45 (2123)	37 (2106)	36 (2243)
**AMI**	11 (2269)	7 (1725)	5 (1821)	5 (1954)	6 (2147)	4 (2070)	3 (1985)	2 (1976)	2 (1926)	1 (2044)
**CIP**	40 (2275)	42 (1725)	37 (1821)	40 (1958)	48 (2147)	52 (2072)	54 (1988)	54 (1978)	49 (1927)	46 (2048)
**COL**	1 (2538)	5 (1868)	5 (2017)	4 (2179)	3 (2337)	2 (2247)	2 (2187)	1 (2122)	3 (2102)	1 (2229)
**TIG**	4 (2254)	6 (1725)	9 (1821)	12 (1958)	8 (2150)	11 (2070)	8 (1985)	5 (1957)	7 (1929)	8 (2041)

Legend: Resistance percentages (total number of isolates tested).

**Table 5:** Resistance of *Pseudomonas aeruginosa* to antibiotics at the University Hospital Olomouc in 2010–2019.

	2010	2011	2012	2013	2014	2015	2016	2017	2018	2019
**PPT**	8 (1360)	23 (1353)	24 (1627)	30 (1677)	26 (1529)	28 (1664)	17 (1689)	13 (1541)	13 (1472)	14 (1725)
**CTZ**	18 (1367)	19 (1353)	15 (1625)	24 (1677)	18 (1529)	18 (1664)	12 (1689)	9 (1541)	13 (1472)	17 (1724)
**MER**	28 (1367)	39 (1353)	36 (1627)	40 (1677)	36 (1529)	39 (1664)	37 (1688)	32 (1541)	28 (1471)	34 (1724)
**GEN**	22 (1367)	23 (1350)	22 (1626)	26 (1678)	25 (1529)	25 (1664)	20 (1689)	16 (1540)	16 (1471)	11 (1722)
**AMI**	1 (1367)	5 (1352)	4 (1627)	4 (1677)	4 (1528)	5 (1664)	7 (1689)	5 (1541)	6 (1470)	5 (1725)
**CIP**	34 (1366)	35 (1353)	34 (1627)	38 (1675)	34 (1528)	32 (1664)	26 (1689)	24 (1540)	24 (1471)	23 (1725)
**COL**	0 (1043)	0 (1349)	0 (1625)	0 (1678)	0 (1529)	0 (1664)	1 (1689)	0 (1538)	1 (1469)	1 (1725)

Legend: Resistance percentages (total number of isolates tested), CTZ—ceftazidime.

**Table 6:** Resistance of *Staphylococcus aureus* to antibiotics at the University Hospital Olomouc in 2010–2019.

	2010	2011	2012	2013	2014	2015	2016	2017	2018	2019
**OXA**	3 (2129)	4 (1744)	3 (1794)	4 (1825)	3 (1860)	4 (2031)	4 (2111)	6 (2149)	4 (2559)	4 (2615)
**CIP**	5 (2125)	5 (1746)	5 (1794)	6 (1825)	7 (1860)	7 (2029)	7 (2110)	8 (2150)	7 (2560)	7 (2615)
**GEN**	4 (2106)	8 (1739)	11 (1792)	8 (1826)	10 (1858)	8 (2005)	6 (2100)	6 (2148)	6 (2560)	7 (2608)
**VAN**	0 (2127)	0 (1742)	0 (1793)	0 (1822)	0 (1856)	0 (2002)	0 (2072)	0 (2146)	0 (2556)	0 (2610)

Legend: Resistance percentages (total number of isolates tested), OXA—oxacillin, VAN—vancomycin.

**Table 7:** Antibiotic consumption in defined daily doses (DDDs) at the University Hospital Olomouc.

Antibiotic Class/Antibiotic	2010	2011	2012	2013	2014	2015	2016	2017	2018	2019
**Penicillins combined with beta-lactamase inhibitors**	89,977	80,212	77,168	76,803	76,937	81,889	70,248	71,774	74,446	76,427
**3rd and 4th generation cephalosporins**	4497	4056	3713	4018	4188	4601	4812	5553	6250	7716
**Carbapenems**	4518	5216	6223	6761	9956	10,242	10,910	12,322	13,196	11,900
**Aminoglycosides**	8433	10,636	10,695	10,657	9937	10,911	11,517	11,208	10,756	10,413
**Fluoroquinolones**	11,322	10,870	10,618	11,365	11,935	13,642	14,875	12,957	13,133	12,421
**Colistin**	714	1153	1261	1738	1905	2278	1648	1714	1832	1669
**Glycopeptides**	2921	2464	3026	3167	4578	4048	4012	3322	3152	3088
**Tigecycline**	554	572	426	499	1314	1302	2334	2683	3019	2824

**Table 8:** Resistance of *Escherichia coli* to antibiotics at the Department of Anesthesiology and Intensive Care Medicine in 2010–2019.

	2010	2011	2012	2013	2014	2015	2016	2017	2018	2019
**AMS**	40 (115)	48 (143)	49 (140)	28 (129)	45 (110)	42 (103)	37 (97)	45 (102)	44 (116)	34 (182)
**PPT**	13 (116)	21 (141)	20 (138)	16 (125)	15 (107)	23 (100)	22 (98)	25 (102)	27 (117)	25 (182)
**CTX**	9 (116)	16 (141)	17 (138)	14 (125)	10 (107)	20 (100)	14 (97)	18 (102)	26 (117)	31 (182)
**MER**	0 (116)	0 (141)	0 (138)	0 (125)	0 (107)	0 (100)	0 (98)	0 (102)	0 (117)	0 (182)
**GEN**	6 (116)	20 (143)	16 (140)	13 (129)	12 (110)	11 (103)	12 (97)	13 (102)	18 (117)	23 (182)
**AMI**	7 (116)	6 (141)	9 (138)	2 (125)	8 (105)	3 (100)	5 (98)	5 (102)	1 (117)	2 (182)
**CIP**	35 (116)	30 (141)	27 (138)	27 (125)	29 (107)	24 (100)	30 (97)	34 (102)	36 (117)	29 (182)
**COL**	0 (116)	3 (142)	1 (140)	0 (129)	0 (110)	1 (103)	0 (98)	0 (102)	0 (116)	0 (182)
**TIG**	0 (115)	2 (140)	0 (138)	4 (125)	0 (107)	8 (100)	0 (97)	1 (102)	2 (117)	3 (182)

Legend: Resistance percentages (total number of isolates tested).

**Table 9:** Resistance of *Klebsiella pneumoniae* to antibiotics at the Department of Anesthesiology and Intensive Care Medicine in 2010–2019.

	2010	2011	2012	2013	2014	2015	2016	2017	2018	2019
**AMS**	72 (148)	78 (181)	75 (165)	66 (247)	70 (234)	77 (222)	77 (145)	76 (127)	80 (196)	76 (404)
**PPT**	58 (149)	67 (181)	61 (165)	65 (247)	62 (233)	73 (222)	71 (145)	65 (126)	67 (195)	69 (401)
**CTX**	49 (149)	64 (181)	58 (165)	62 (247)	60 (234)	74 (222)	71 (145)	63 (126)	74 (195)	78 (404)
**MER**	0 (149)	3 (181)	0 (165)	<1 (247)	<1 (233)	0 (222)	2 (145)	2 (125)	1 (195)	1 (402)
**GEN**	46 (149)	64 (181)	54 (165)	63 (247)	60 (233)	75 (222)	72 (145)	60 (127)	62 (195)	71 (402)
**AMI**	19 (149)	14 (181)	9 (165)	13 (246)	17 (233)	6 (222)	6 (145)	4 (126)	2 (195)	7 (402)
**CIP**	64 (149)	71 (181)	53 (165)	63 (247)	64 (233)	76 (222)	75 (145)	67 (126)	77 (195)	72 (402)
**COL**	3 (148)	13 (181)	10 (165)	8 (247)	9 (233)	2 (222)	7 (145)	2 (127)	6 (195)	1 (398)
**TIG**	7 (148)	9 (181)	13 (165)	12 (247)	7 (234)	8 (222)	17 (145)	6 (126)	12 (195)	7 (403)

Legend: Resistance percentages (total number of isolates tested).

**Table 10:** Resistance of *Pseudomonas aeruginosa* to antibiotics at the Department of Anesthesiology and Intensive Care Medicine in 2010–2019.

	2010	2011	2012	2013	2014	2015	2016	2017	2018	2019
**PPT**	29 (106)	32 (150)	39 (188)	41 (200)	45 (224)	42 (223)	46 (150)	44 (111)	39 (142)	43 (219)
**CTZ**	32 (106)	35 (150)	39 (188)	41 (200)	48 (224)	42 (223)	35 (150)	34 (111)	31 (142)	33 (218)
**MER**	22 (106)	31 (150)	33 (188)	38 (200)	36 (224)	44 (223)	52 (149)	53 (111)	56 (142)	59 (219)
**GEN**	31 (106)	42 (150)	44 (188)	37 (200)	36 (224)	43 (223)	36 (150)	26 (109)	33 (141)	32 (217)
**AMI**	0 (106)	5 (149)	5 (188)	2 (200)	5 (224)	7 (223)	9 (150)	12 (111)	16 (141)	3 (219)
**CIP**	29 (106)	33 (150)	48 (188)	47 (200)	41 (224)	43 (223)	40 (150)	35 (111)	37 (142)	35 (219)
**COL**	0 (83)	0 (150)	0 (188)	0 (200)	0 (224)	0 (223)	0 (150)	0 (109)	2 (142)	1 (217)

Legend: Resistance percentages (total number of isolates tested).

**Table 11:** Resistance of *Staphylococcus aureus* to antibiotics at the Department of Anesthesiology and Intensive Care Medicine in 2010–2019.

	2010	2011	2012	2013	2014	2015	2016	2017	2018	2019
**OXA**	9 (46)	5 (37)	9 (56)	14 (65)	6 (47)	16 (45)	5 (43)	6 (48)	12 (49)	5 (83)
**CIP**	11 (46)	8 (37)	7 (56)	20 (65)	4 (47)	18 (45)	7 (43)	6 (48)	16 (49)	11 (83)
**GEN**	0 (45)	0 (37)	13 (56)	5 (65)	6 (47)	5 (43)	2 (43)	6 (48)	4 (49)	11 (83)
**VAN**	2 (46)	0 (37)	0 (56)	0 (65)	0 (47)	0 (43)	0 (42)	0 (48)	0 (48)	0 (83)

Legend: Resistance percentages (total number of isolates tested).

**Table 12:** Antibiotic consumption in defined daily doses (DDD) at the Department of Anesthesiology and Intensive Care Medicine.

Antibiotic Class/Antibiotic	2010	2011	2012	2013	2014	2015	2016	2017	2018	2019
**Penicillins combined with beta-lactamase inhibitors**	1539	1463	1369	1376	1357	1288	1486	1519	1339	1473
**3rd and 4th generation cephalosporins**	125	130	234	144	428	241	260	325	394	556
**Carbapenems**	618	739	505	946	1290	1427	1298	1280	1498	1822
**Aminoglycosides**	209	691	629	667	682	806	812	589	677	841
**Fluoroquinolones**	589	460	514	576	501	639	670	484	398	510
**Colistin**	167	253	233	410	433	498	340	190	228	478
**Glycopeptides**	83	75	128	204	191	237	324	158	148	249
**Tigecycline**	55	85	80	70	185	230	245	275	450	415

## Data Availability

Data sharing not applicable.
